# Inheritance of Hetero-Diploid Pollen *S*-Haplotype in Self-Compatible Tetraploid Chinese Cherry (*Prunus pseudocerasus* Lindl)

**DOI:** 10.1371/journal.pone.0061219

**Published:** 2013-04-15

**Authors:** Chao Gu, Qing-Zhong Liu, Ya-Nan Yang, Shu-Jun Zhang, Muhammad Awais Khan, Jun Wu, Shao-Ling Zhang

**Affiliations:** 1 College of Horticulture, Nanjing Agricultural University, Nanjing, Jiangsu, People’s Republic of China; 2 Key Laboratory of Plant Germplasm Enhancement and Specialty Agriculture, Wuhan Botanical Garden, Chinese Academy of Sciences, Wuhan, Hubei, People’s Republic of China; 3 Key Laboratory for Fruit Biotechnology Breeding of Shandong, Pomological Institute of Shandong, Taian, Shandong, People’s Republic of China; 4 Department of Natural Resource and Environmental Sciences, University of Illinois at Urbana-Champaign, Urbana, Illinois, United States of America; United States Department of Agriculture, United States of America

## Abstract

The breakdown of self-incompatibility, which could result from the accumulation of non-functional *S*-haplotypes or competitive interaction between two different functional *S*-haplotypes, has been studied extensively at the molecular level in tetraploid Rosaceae species. In this study, two tetraploid Chinese cherry (*Prunus pseudocerasus*) cultivars and one diploid sweet cherry (*Prunus avium*) cultivar were used to investigate the ploidy of pollen grains and inheritance of pollen-*S* alleles. Genetic analysis of the *S*-genotypes of two intercross-pollinated progenies showed that the pollen grains derived from Chinese cherry cultivars were hetero-diploid, and that the two *S*-haplotypes were made up of every combination of two of the four possible *S*-haplotypes. Moreover, the distributions of single *S*-haplotypes expressed in self- and intercross-pollinated progenies were in disequilibrium. The number of individuals of the two different *S*-haplotypes was unequal in two self-pollinated and two intercross-pollinated progenies. Notably, the number of individuals containing two different *S*-haplotypes (*S_1_*- and *S_5_*-, *S_5_*- and *S_8_*-, *S_1_*- and *S_4_*-haplotype) was larger than that of other individuals in the two self-pollinated progenies, indicating that some of these hetero-diploid pollen grains may have the capability to inactivate stylar S-RNase inside the pollen tube and grow better into the ovaries.

## Introduction

*Prunus* species are reported to show gametophytic self-incompatibility (GSI) [1], a widespread mechanism in flowering plants that prevents inbreeding and promotes outcrossing [2]. GSI is controlled by a single multi-allelic locus, termed the *S*-locus. The *S*-locus contains at least two genes, the pistil determinant (stylar-*S*) and the pollen determinant (pollen-*S*). The stylar-*S* gene is an *S-RNase* discovered in the Rosaceae [3], [4], Solanaceae [5], [6] and Plantaginaceae [7], is expressed in the pistil, and specifically degrades the RNA of incompatible pollen [8]. The pollen-*S* gene, recently identified in the Rosaceae [9]–[12], Solanaceae [13], [14] and Plantaginaceae [15], is an F-box pollen-expressed gene (*S*-locus *F-box* [*SLF*]/*S*-haplotype specific *F-box* [*SFB*]) that is tightly linked to the *S-RNase* allele. The C-terminal region of the F-box protein functions as a receptor to incorporate a target protein into an SCF (Skp1, Cdc53/Cullin, F-box receptor) complex for polyubiquitination and eventual degradation by the 26S proteasome [16]. These C-terminal variable regions may also be responsible for the discrimination between self and non-self S-RNases [9].

The breakdown of self-incompatibility can result from stylar-*S* mutations [17]–[19], pollen-*S* mutations [20]–[24], polyploidy [25]–[31] or other modifying factors [32], [33]. Although it has been reported in *Nicotiana* [34], [35], *Antirrhinum* [36], *Petunia* [13], [37], [38], *Lycopersicon* [39], [40], *Trifolium* [41], *Oenothera* [42], *Pyrus* [29], [43] and *Prunus* species [28], [44] that polyploidy and duplication of the pollen-*S* allele can also break down self-incompatibility, not all polyploid cultivars are self-compatible, for example, cultivars that contain a maximum of one non-functional *S*-haplotypes are self-incompatible. Other cultivars containing a minimum of two non-functional *S*-haplotypes are self-compatible in tetraploid *Prunus cerasus* [27], [30].

Competitive interaction is a well-known phenomenon that could explain the breakdown of self-incompatibility in tetraploid cultivars [38], [45], [46]. It has been confirmed that hetero-allelic diploid pollen broke down the self-incompatibility in tetraploid cultivar ‘Sha01’ (*Pyrus sinkiangensis* Yü) [29] and tetraploid Chinese cherry [28]. As only one Chinese cherry cultivar, “Nanjing Chuisi”, was used in the studies, the support of competitive interaction in *Prunus* in general is still incomplete [47]. Thus, in this study, two other tetraploid Chinese cherry cultivars were used to characterize the *S*-genotypes of pollen grains and the inheritance of *S*-haplotypes. The results obtained will enhance the current understanding of competitive interaction and give details of the self-incompatibility reaction.

## Materials and Methods

### Plant Material

Two self-compatible tetraploid Chinese cherry cultivars were collected: “Dabai” (*S_1_S_2_S_5_S_8_*) [44] was provided by Tian-Qi Chen from the town of Taihe in Anhui province, China; “Taishanganying” (*S_1_S_2_S_4_S_6_*) [44] and a sweet cherry cultivar “Summit” (*S_1_S_2_*) [48] were provided by Qing-Zhong Liu from Pomological Institute of Shandong, China. The ploidy level of all cultivars was detected by flow cytometry.

The flowers of all cherry cultivars were collected immediately before anthesis, and the styles and pollen grains were detached and stored in liquid nitrogen until use. Four progenies and 961 seedlings derived from self-pollination of “Dabai” and “Taishanganying”, and the interspecific crosses “Summit”×“Dabai” and “Summit” × “Taishanganying” were used in this study (Table S1 in File S1).

Plant species from which each sequence is derived are represented by their initials: *Prunus pseudocerasus (Pps), Prunus avium (Pa), Prunus armeniaca (Par), Prunus dulcis (Pd), Prunus mume (Pm), Prunus salicina (Ps), Prunus speciosa (Pspe), Prunus spinosa (Pspi)*.

### Isolation of Nucleic Acids

Genomic DNA of all three cherry cultivars and progeny plants was isolated from young leaves by the Cetyl Trimethyl Ammonium Bromide (CTAB)-based extraction method, with modifications [49]. Homogenates were treated with RNase I (Invitrogen, CA, USA) and incubated at 37°C for 1 h. Total RNA was extracted from pollen grains, styles and leaves of the three cultivars and treated with DNase I (Invitrogen) [28]. Total RNA (1 µg) from isolated styles, pollen and leaves was used for first-strand cDNA synthesis using the RNA PCR Kit Version 2.1 (TaKaRa, Kyoto, Japan) with the Adp-dT primer set, which consisted of the M13-20 sequence primer and oligo(dT)_16_ [50]. The cDNAs then served as templates for subsequent PCR amplification.

### RT-PCR and CAPS Markers for Discriminating Different *S-RNase* and *SFB* Genes

The primers Pru-C2 and Pa-C5R were used to amplify the sequences of *S-RNase* alleles (Table S2 in File S1). The PCR reaction mix and cycling conditions were as described [44]. The primers PsSFB-F1 and PsSFB-R1 were used to amplify the sequences of *SFB* alleles (Table S2 in File S1). The PCR reaction mix and cycling conditions were as described [12].

CAPS markers are reliable tools for discriminating PCR fragments of similar size [51]. In the present study, the cDNA amplification products using two primer pairs had only one band, but contained several different *S-RNase* or *SFB* alleles. CSP6I (Invitrogen) was used to digest PCR products of stylar cDNA, and CSP6I (Invitrogen), DpnII, HpyCH4III, HpyCH4IV, BslI and BsaJI (NEB, Mass., USA) were used to digest PCR products of pollen cDNA. The digested products were analyzed on 2% agarose gels run in 1×TAE buffer and visualized under UV light.

The full-length *S-RNases* and *SFBs* were obtained by 5′ and 3′ rapid-amplification of cDNA ends (5′- and 3′-RACE). The PCR mixture and thermal cycling conditions were as described in GeneRacer™ Kit (Invitrogen).

### Organ-specific Expression and DNA Walking

Stylar, pollen grain and leaf cDNAs of “Dabai” and “Taishanganying” were used as templates for PCR amplification with primers PsSFB-F1 and PsSFB-R1 for *SFB* alleles. PCR analysis of *actin* genes using the primers ActF and ActR was used as the internal control. Genomic DNA was used as another control.

Three specific primers (GC-C3F-1, GC-C3F-2 and Pa-C4F) were designed from the conserved sequence of *S-RNase* alleles (Table S2 in File S1), and two specific primers (Mid-SFB-1 and Mid-SFB-2) were designed from the conserved sequence of *SFB* alleles for DNA walking (Table S2 in File S1). The PCR reaction mixture and cycling conditions were as described in the Genome Walking Kit (TaKaRa). PCR products were analyzed on 1.5% agarose gels run in 1×TAE buffer and visualized under UV light.

### Cloning and Sequencing

Specific PCR fragments were excised from 2% agarose gels and purified using the Qiagen Gel Extraction Kit (Qiagen, Valencia, Calif., USA). The purified products were cloned into the PMD19-T vector (TaKaRa) following the manufacturer’s instructions and transformed into *E. coli* DH5α. Transformed colonies were selected and plasmid purification was performed using the Plasmid Mini Kit (Qiagen). Plasmids with inserts of the expected size were examined using the same primer pairs used for initial amplification by PCR. To obtain a consensus sequence and to avoid errors caused by PCR, three independent positive clones of each *S*-haplotype were sequenced by Invitrogen.

### Sequence Analysis of *S-RNase* and *SFB* Alleles

A consensus DNA sequence for each allele was obtained by assembling the data from all three replicates with DNAMAN (version 5.2; Lynnon Biosoft). The deduced amino acid sequences of the *S-RNases* and *SFBs* were aligned using the CLUSTAL W [52] and CLUSTAL X [53] programs, respectively.

GenBank accession numbers of genes mentioned in this article were as follows: *Pps-S_1_* (HQ913630), *Pps-S_2_* (FJ543097), *Pps-S_4_* (FJ543098), *Pps-S_5_* (HQ913631), *Pps-S_6_* (FJ543099), *Pps-S_8_* (HQ913635), *Pa-S_1_* (AB028153), *Pa-S_2_* (AJ298311), *Pm-S_3_* (AB376969), *Par-S_1_* (AY587561), *Pps-SFB_1_* (HQ913632), *Pps-SFB_4_* (HQ913633), *Pps-SFB_5_* (EU253964), *Pps-SFB_6_* (HQ913634), *Pa-SFB_1_* (AB111518), *Pa-SFB_2_* (AB111519), *Par-SFB_1_* (AY587563), *Pd-SFB_b_* (AB092967), *Pm-SFB_7_* (AB101441), and *Ps-SFB_c_* (AB280792).

### Field Pollination Tests and Genetic Segregation of *S*-alleles

The total number of self-pollinated flowers was 5212 for “Dabai”, 3692 for “Taishanganying” and 1073 for “Summit”; the number of cross-pollinated flowers was 2815 for “Summit”×“Dabai” and 2106 for “Summit”×“Taishanganying” (Table S1 in File S1). The emasculated flowers were self−/cross- pollinated, counted and re-bagged at full bloom. The percentages of fruit set were counted before harvest with ≥2% considered to be self- or cross-compatible [54].

To study the segregation of *S*-haplotypes, seedlings were genotyped by PCR amplification with primers Pru-C2 and Pa-C3R for *S-RNases*. The second intron in the *S_8_-RNase* allele was too large to amplify successfully, thus the specific reverse primer Pps-S8R was used in this study (Table S2 in File S1). The chi squared (χ^2^) goodness-of-fit was applied to the genotyping data to test for deviation from expected Mendelian segregation ratios. Moreover, to test the genetic relationship between *SFBs* and *S-RNases* in tetraploid Chinese cherry cultivars, PCR was performed using DNA extracted from individuals as a template with primers PsSFB-F1 and PsSFB-R1; genomic DNA from the parent(s) was used for the control. The amplification products were digested with Csp6I.

## Results

### Isolation of *S-RNase* and *SFB* Alleles

The full-length sequences of six *S-RNase* alleles were obtained by RT-PCR in combination with CAPS markers (Csp6I) from the styles of two tetraploid Chinese cherry cultivars, “Dabai” (*S_1_S_2_S_5_S_8_*) and “Taishanganying” (*S_1_S_2_S_4_S_6_*). However, only four *SFB* alleles were isolated by RT-PCR from the pollen of the two cultivars, although six restriction endonucleases (Csp6I, DpnII, HpyCH4III, HpyCH4IV, BslI and BsaJI) were used to digest the PCR amplification products from the recombined plasmid. Two digested patterns of *SFB_1_* and *SFB_5_* were found in “Dabai” (Figure S1 in File S1) and three patterns of *SFB_1_*, *SFB_4_* and *SFB_ 6_* were found in “Taishanganying” (Figure S2 in File S1). No pattern of *SFB_8_* was found in “Dabai” and *SFB_2_* was not detected in either cultivar suggesting that they may have a low expression level, differential amplification, or a large sequence insertion and/or deletion in coding region.

### Sequence Analyses and Comparison of *S-RNase* and *SFB* Alleles

**Figure 1:**
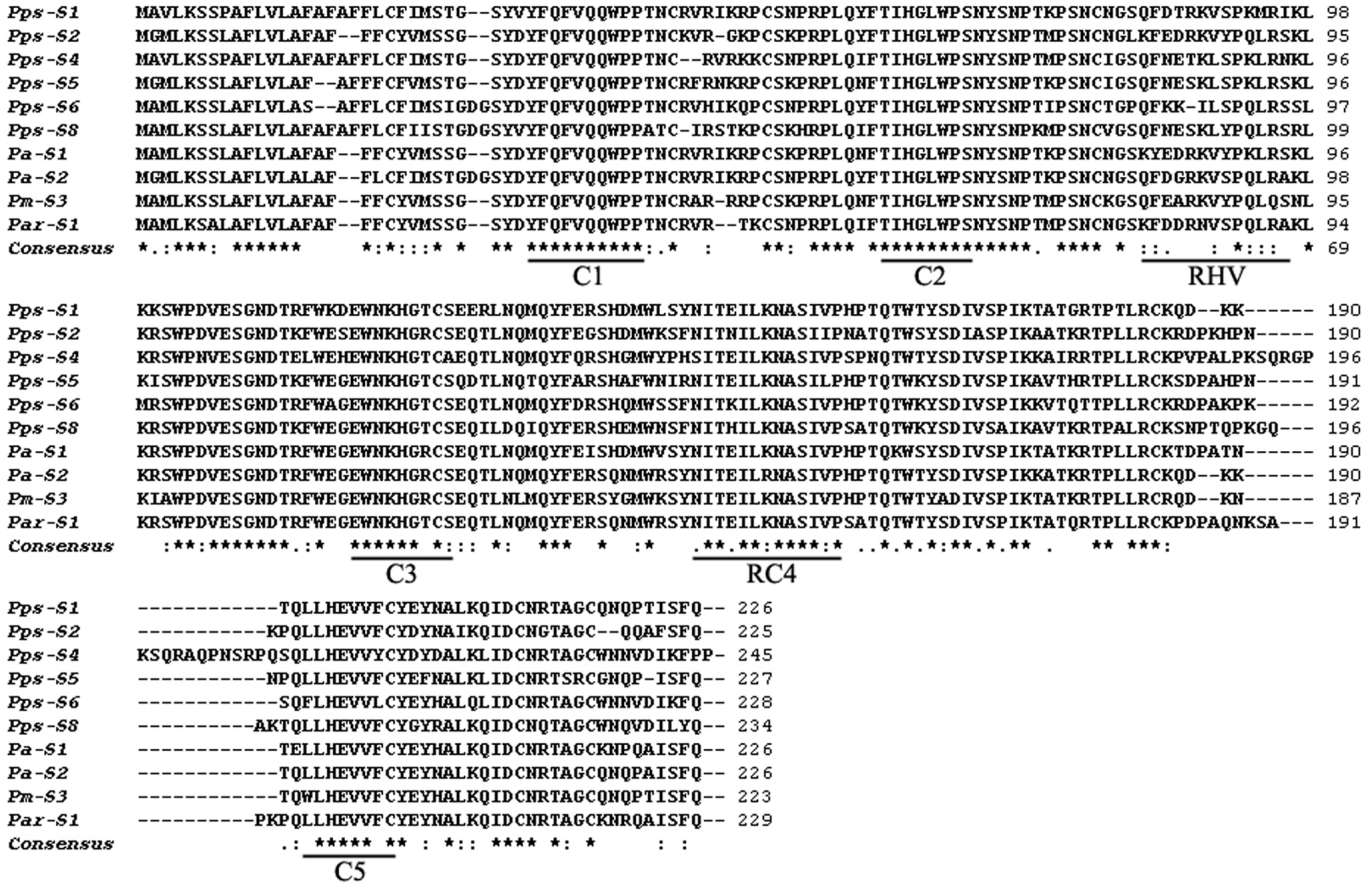
Alignment of the deduced amino acid sequences of *S-RNases* from Chinese cherry and other *Prunus* species. *Asterisks, dot,* and *dashes* indicate conserved amino acid sites, conservative substitutions, and gaps, respectively. The five conserved (C1, C2, C3, RC4 and C5) and hypervariable (RHV) regions are *underlined*.

The alignment of the deduced amino acid sequences showed that the six *PpsS-RNase* alleles contained five conserved regions (C1, C2, C3, RC4 and C5) and one hypervariable region (RHV; Figure 1) that is crucial for determining the *S*-allele specificity for *S-RNase* alleles [55]. The deduced amino acid identities ranged from 70.5% (*Pps-S_2_*/*Pps-S_4_*) to 79.4% (*Pps-S_1_*/*Pps-S_5_*) among six *PpsS-RNases*; exceptionally high identities were detected when compared with other *Prunus* species, for example, the identity was 99.4% between *Pps-S_1_* and *Pspe-S_46_*, 99.1% between *Pps-S_5_* and *Pspi-S_16_*, and 100% between *Pps-S_8_* and *Pspe-S_35_*.

**Figure 2:**
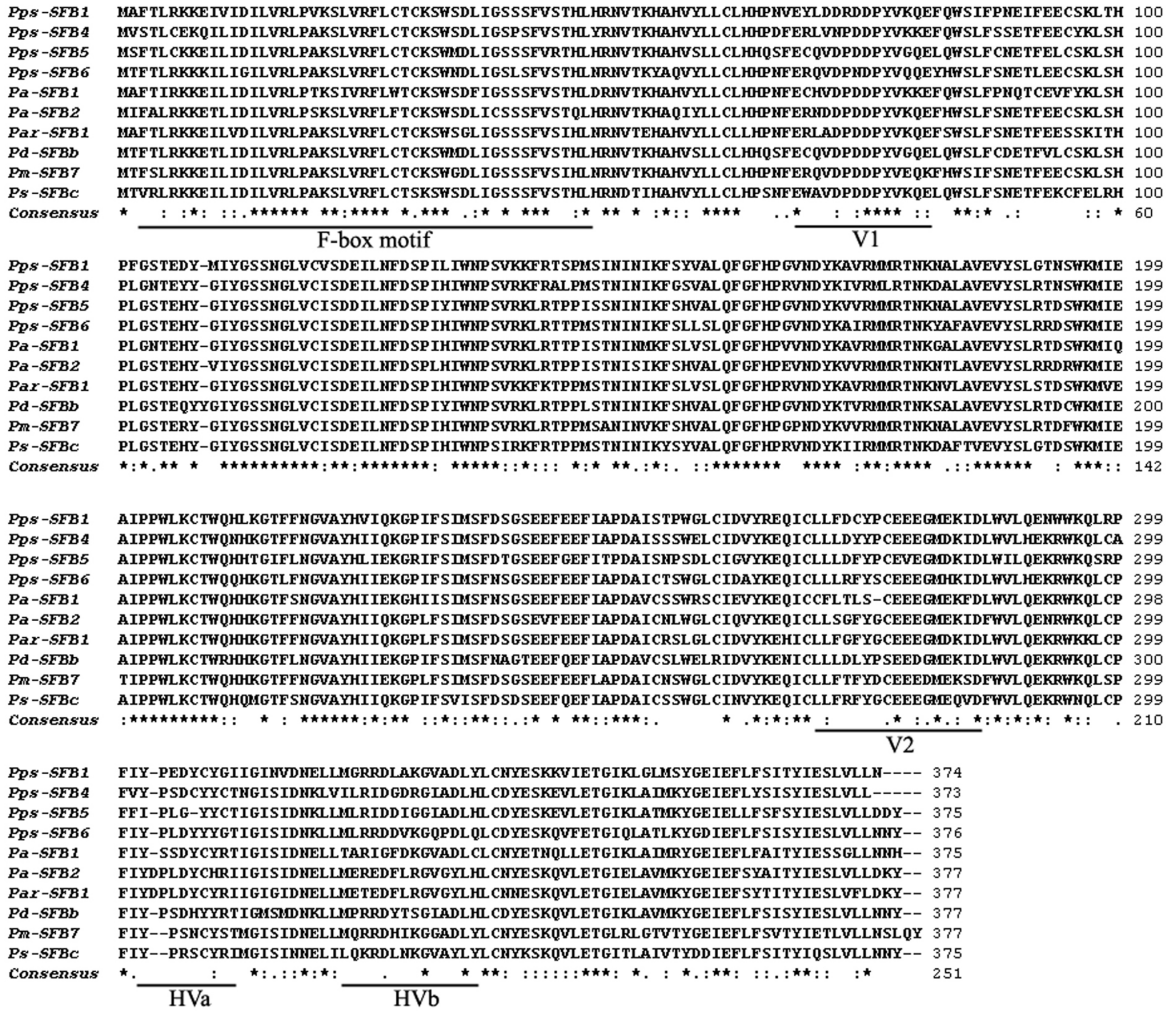
Alignment of the deduced amino acid sequences of *SFBs* from Chinese cherry and other *Prunus* species. *Asterisks, dot,* and *dashes* indicate conserved amino acid sites, conservative substitutions, and gaps, respectively. F-box motif, variable (V1 and V2) and hypervariable (HVa and HVb) regions are *underlined*.

The alignment of the deduced amino acid sequences showed that the four *PpsSFB* alleles contained one conserved F-box motif, two variable regions (V1 and V2), and two hypervariable regions (HVa and HVb; Figure 2). The amino acid identities ranged from 76.9% (*Pps-SFB_1_*/*Pps-SFB_5_*) to 80% (*Pps-SFB_4_*/*Pps-SFB_5_*); again, high identities were detected when compared with other *Prunus SFBs*. For example, the identity was 96.8% between *Pps-SFB_1_* and *Pm-SLF_9_*, and 96.8% between *Pps-SFB_5_* and *Pspi-SFB_16_*.

### Organ Specific Expression of *SFB*s

**Figure 3:**
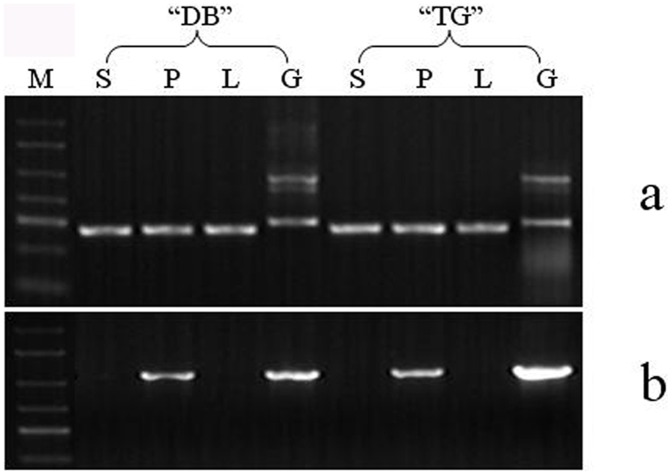
Expression patterns of *SFBs* in two cultivars of Chinese cherry. RT-PCR was performed using total RNA with primers to detect transcripts of *SFB* and *Actin* genes. S, P and L stand for style, pollen and leaf RNA with reverse transcriptase, respectively; G is genomic DNA and M is ladder marker. a. PCR amplification with primers ActF and ActR; b. PCR amplification with primers PsSFB-F1 and PsSFB-R1.

Total RNAs extracted from the leaf, style and pollen grains in “Dabai” and “Taishanganying” were amplified by RT-PCR with ActF and ActR primers. The resulting DNA fragments were similar in size but were shorter than those amplified from genomic DNA because of the lack of introns (Figure 3a), confirming successful synthesis of first-strand cDNA and the absence of genomic DNA. Using the gene-specific primers PsSFB-F1 and PsSFB-R1 for *SFBs,* only RT-PCR of pollen RNAs, but not of style and leaves, produced fragment(s) of the same size as those produced by PCR of genomic DNA (Figure 3b). These results indicate that the *SFB* alleles isolated from the pollen grains of two cultivars were expressed specifically in pollen.

### Physical Distance between *S-RNase* and *SFB* Alleles

**Figure 4:**
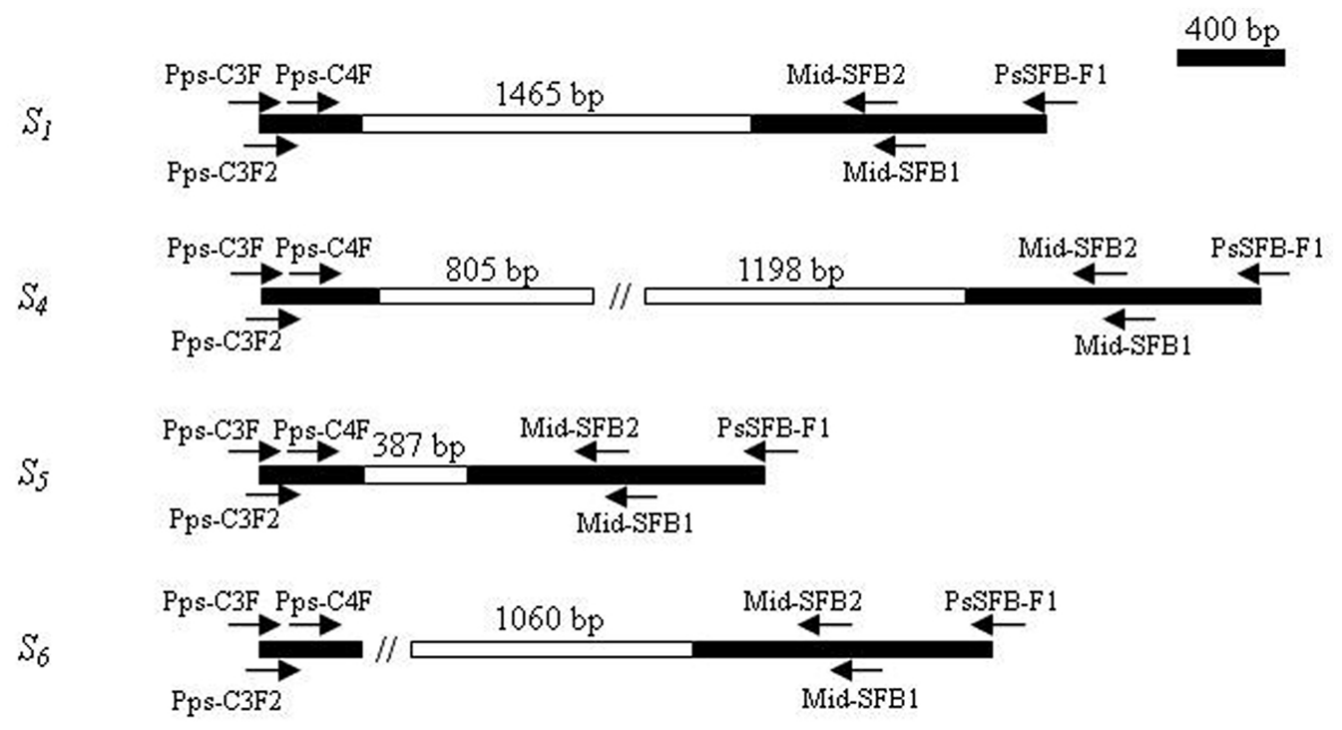
Schematic representation of the *S* locus of four *S*-haplotypes. Black and white boxes represent exons and intergenic region, respectively. Double slashes show the regions which have not been determined in this study. The positions of the primers (Table S2 in File S1) used in the present study are indicated by arrows.

To confirm the transcription direction and linkage relationship of the *S-RNases* and *SFB* alleles in *P. pseudocerasus*, DNA walking was performed using a series of specific primers for each *S* allele (Table S2 in File S1). The distances between two genes in the *S_1_*- and *S_5_*-haplotypes were 1465 bp and 387 bp, respectively; the transcription of the *SFB_1_* and *SFB_5_* alleles were in the reverse direction of their *S-RNases* (Figure 4). Thus, *SFB* alleles in *P. pseudocerasus* were physically linked, with opposite transcription orientations for their *S-RNases*. However, the intergenic sequences of the *S_4_*- and *S_6_*-haplotypes were incomplete (Fig. 4), perhaps due to their great length, as found for the *S_4_-*haplotype of *P. avium* (i.e., 40 kb) [56].

### Genetic Analysis of the Self-compatible Cultivar “Dabai”

The genotypic ratio of the progeny of “Summit”×“Dabai” did not fit the theoretical segregation ratio of 1∶1:1∶1:1∶1:1∶1:1∶1:1∶1 (χ^2^ = 38.918>χ^2^_0.05, 11_ = 19.68; Table 1, Table S3 in File S1), but the segregation ratio of the two *S*-alleles in “Summit”, *Pa-S_1_*: *Pa-S_2_* was 25∶24 (Figure S3 in File S1), which fit the theoretical ratio of 1∶1 (χ^2^ = 0.02<χ^2^_0.05, 1_ = 3.84; Table 2, Table S3 in File S1). The segregation results showed that only the *S*-haplotypes expressed in the pollen grains of *S_1_S_2_*, *S_1_S_5_*, *S_1_S_8_* and *S_5_S_8_* could be transmitted to the progeny, and single, ternary and/or quaternary *S*-alleles derived from “Dabai”, such as *S_1_*, *S_2_*, *S_5_*, *S_8_*, *S_1_S_2_S_5_*, *S_1_S_2_S_8_*, *S_1_S_5_S_8_*, *S_2_S_5_S_8_* and *S_1_S_2_S_5_S_8_*, were not found in this progeny (Figure S3 in File S1). However, the individuals of *S_2_S_5_* and *S_2_S_8_* could not be found in the progeny (Figure S4 in File S1). The ratio of *S_1_S_2_*, *S_1_S_5_*, *S_1_S_8_*, *S_5_S_8_*, *S_2_S_5_* and *S_2_S_8_* was approximately 1∶2:2∶2:0∶0 (χ^2^ = 0.357<χ^2^_0.05, 5_ = 7.815; Table 2), which did not fit the theoretical ratio of 1∶1:1∶1:1∶1 (χ^2^ = 31.204>χ^2^_0.05, 5_ = 7.815; Table 2, Table S3 in File S1).

**Table 1:** Segregation of *S*-genotypes in different self- or cross-pollinated progeny plants.

Self- or Cross- pollination	*S*-genotypes observed in progeny												Total	Expected ratios	?^2^	?^2^_0.05_ *
‘Summit’×‘Daiba’	*S_1_S_2_*/*Pa-S_1_*	*S_1_S_2_*/*Pa-S_2_*	*S_1_S_5_*/*Pa-S_1_*	*S_1_S_5_*/*Pa-S_2_*	*S_1_S_8_*/*Pa-S_1_*	*S_1_S_8_*/*Pa-S_2_*	*S_2_S_5_*/*Pa-S_1_*	*S_2_S_5_*/*Pa-S_2_*	*S_2_S_8_*/*Pa-S_1_*	*S_2_S_8_*/*Pa-S_2_*	*S_5_S_8_*/*Pa-S_1_*	*S_5_S_8_*/*Pa-S_2_*	49	1∶1:1∶1:1∶1:1∶1:1∶1:1∶1	38.918	19.68
	2	4	8	5	10	5	0	0	0	0	5	10				
‘Summit’×‘Tianshanganying’	*S_1_S_2_*/*Pa-S_1_*	*S_1_S_2_*/*Pa-S_2_*	*S_1_S_4_*/*Pa-S_1_*	*S_1_S_4_*/*Pa-S_2_*	*S_1_S_6_*/*Pa-S_1_*	*S_1_S_6_*/*Pa-S_2_*	*S_2_S_4_*/*Pa-S_1_*	*S_2_S_4_*/*Pa-S_2_*	*S_2_S_6_*/*Pa-S_1_*	*S_2_S_6_*/*Pa-S_2_*	*S_4_S_6_*/*Pa-S_1_*	*S_4_S_6_*/*Pa-S_2_*	24	1∶1:1∶1:1∶1:1∶1:1∶1:1∶1	19	19.68
	2	4	5	1	5	1	0	0	0	1	2	3				
‘Daiba’ self-fertility	*S_1_S_2_*	*S_1_S_5_*	*S_1_S_8_*	*S_2_S_5_*	*S_2_S_8_*	*S_5_S_8_*	*S_1_S_2_S_5_*	*S_1_S_2_S_8_*	*S_1_S_5_S_8_*	*S_2_S_5_S_8_*	*S_1_S_2_S_5_S_8_*		438	1∶1:1∶1:1∶1:6∶6:6∶6:6	1122.315	18.31
	0	90	4	0	0	53	14	6	211	0	60					
‘Tianshanganying’ self-fertility	*S_1_S_2_*	*S_1_S_4_*	*S_1_S_6_*	*S_2_S_4_*	*S_2_S_6_*	*S_4_S_6_*	*S_1_S_2_S_4_*	*S_1_S_2_S_6_*	*S_1_S_4_S_6_*	*S_2_S_4_S_6_*	*S_1_S_2_S_4_S_6_*		450	1∶1:1∶1:1∶1:6∶6:6∶6:6	349.253	18.31
	0	41	9	0	0	0	124	13	133	3	127					

* Degree of freedom.

**Table 2:** Segregation of *S*-alleles and diploid pollen-*S* in progeny of different self- or cross-pollinations.

Self- or Cross- pollination	*S*-alleles observed in progeny						Segregation ratios (approximation)	?^2^	?^2^_0.05_ *
‘Summit’×‘Daiba’	*Pa-S_1_*	*Pa-S_2_*					1∶1^a^	0.02^b^	3.84
	25	24					1∶1^c^	0.02^d^	
	*Pps-S_1_*	*Pps-S_2_*	*Pps-S_5_*	*Pps-S_8_*			1∶1:1∶1^a^	19.388^b^	7.815
	34	6	28	30			5∶1:5∶5^c^	0.612^d^	
	*S_1_S_2_*	*S_1_S_5_*	*S_1_S_8_*	*S_5_S_8_*	*S_2_S_5_*	*S_2_S_8_*	1∶1:1∶1:1∶1^a^	31.204	11.07
	6	13	15	15	0	0	1∶2:2∶2:0∶0	0.357^d^	
‘Summit’×‘Tianshanganying’	*Pa-S_1_*	*Pa-S_2_*					1∶1	0.667^b^	3.84
	14	10					1∶1^c^	0.667^d^	
	*Pps-S_1_*	*Pps-S_2_*	*Pps-S_4_*	*Pps-S_6_*			1∶1:1∶1^a^	5.167^b^	7.815
	18	7	11	12			2∶1:2∶2^c^	2.094^d^	
	*S_1_S_2_*	*S_1_S_4_*	*S_1_S_6_*	*S_2_S_4_*	*S_2_S_6_*	*S_4_S_6_*	1∶1:1∶1:1∶1^a^	9.5^b^	11.07
	6	6	6	0	1	5	6∶6:6∶0:1∶6^c^	0.132^d^	
‘Daiba’ self-fertility	*Pps-S_1_*	*Pps-S_2_*	*Pps-S_5_*	*Pps-S_8_*			1∶1:1∶1^a^	237.923^b^	7.815
	385	80	428	334			5∶1:5∶4^c^	2.472^d^	
	*S_1_S_2_*	*S_1_S_5_*	*S_1_S_8_*	*S_2_S_5_*	*S_2_S_8_*	*S_5_S_8_*	1∶1:1∶1:1∶1^a^	503.97^b^	11.07
	80	375	281	74	66	324	3∶16:12∶3:3∶16^c^	7.556^d^	
‘Tiashanganying’ self-fertility	*Pps-S_1_*	*Pps-S_2_*	*Pps-S_4_*	*Pps-S_6_*			1∶1:1∶1^a^	301.751^b^	7.815
	447	267	428	285			3∶2:3∶2^c^	2.021^d^	
	*S_1_S_2_*	*S_1_S_4_*	*S_1_S_6_*	*S_2_S_4_*	*S_2_S_6_*	*S_4_S_6_*	1∶1:1∶1:1∶1^a^	149.426^b^	11.07
	264	425	282	254	143	263	2∶3:2∶2:1∶2^c^	3.162^d^	

* Degree of freedom,

a Theoretical ratios,

b Theoretical χ^2^ values,

c Expected ratios,

d Expected χ^2^ values.

The results also showed that individuals of *S_1_S_5_*, *S_1_S_8_*, *S_5_S_8_*, *S_1_S_2_S_5_*, *S_1_S_2_S_8_*, *S_1_S_5_S_8_* and *S_1_S_2_S_5_S_8_* were found in self-pollinated progeny of “Dabai”, but individuals with *S_1_S_2_*, *S_2_S_5_*, *S_2_S_8_* and *S_2_S_5_S_8_* were not found (Figure S4 in File S1), and their ratio did not fit the theoretical ratio of 1∶1:1∶1:1∶1:6∶6:6∶6:6 (χ^2^ = 1122.315>χ^2^_0.05, 11_ = 18.31; Table 1, Table S4 in File S1). The ratio of *S_1_S_2_*, *S_1_S_5_*, *S_1_S_8_*, *S_2_S_5_*, *S_2_S_8_* and *S_5_S_8_* was approximately 3∶16:12∶3:3∶16 (χ^2^ = 7.556<χ^2^_0.05, 5_ = 11.07; Table 2), which also did not fit the theoretical ratio of 1∶1:1∶1:1∶1 (χ^2^ = 503.97>χ^2^_0.05, 2_ = 11.07; Table 2). The number of individuals with *S_1_S_5_* and *S_5_S_8_* genotypes was greater than other individuals containing two different *S*-haplotypes, indicating that these two pairs of *S*-haplotypes expressed in pollen grains are able to grow better into ovaries than the pollen grains of *S_1_S_2_*, *S_1_S_8_*, *S_2_S_5_* and *S_2_S_8_*.

The ratio of single *S*-alleles derived from “Dabai”, *S_1_*, *S_2_*, *S_5_* and *S_8_*, was approximately 5∶1:5∶5 (χ^2^ = 0.612<χ^2^_0.05, 3_ = 7.815; Table 2) in the cross-pollinated progeny of “Summit”×“Dabai”, and 5∶1:5∶4 (χ^2^ = 2.472<χ^2^_0.05, 3_ = 7.815; Table 2) in the self-pollinated progeny of “Dabai”, which do not fit the theoretical ratio of 1∶1:1∶1 (Table 2). This result suggests that the hetero-diploid pollen grains containing the *S_2_*-haplotype may not be able to inactivate stylar S-RNase inside the pollen tube.

### Genetic Analysis of the Self-compatible Cultivar of “Taishanganying”

The segregation of *S*-alleles in the progeny of “Summit”×“Taishanganying” was in relative equilibrium. The segregation ratio of this progeny was consistent with the theoretical segregation ratio of 1∶1:1∶1:1∶1:1∶1:1∶1:1∶1 (χ^2^ = 19<χ^2^_0.05, 11_ = 19.68; Table 1, Table S3 in File S1). The segregation ratio of the two *S*-alleles in “Summit”, *Pa-S_1_*: *Pa-S_2_* was 14∶10 (Figure S5 in File S1), which fit the theoretical ratio of 1∶1 (χ^2^ = 0.667<χ^2^_0.05, 1_ = 3.84; Table 2, Table S3 in File S1). The segregation results showed that only the *S*-haplotypes expressed in the pollen grains of *S_1_S_2_*, *S_1_S_4_*, *S_1_S_6_*, *S_2_S_6_* and *S_4_S_6_* could be transmitted to the progeny, and single, ternary and/or quaternary *S*-alleles derived from “Taishanganying”, such as *S_1_*, *S_2_*, *S_4_*, *S_6_*, *S_1_S_2_S_4_*, *S_1_S_2_S_6_*, *S_1_S_4_S_6_*, *S_2_S_4_S_6_* and *S_1_S_2_S_4_S_6_* were not found in the progeny (Figure S5 in File S1). The ratio of the individuals containing two different *S*-haplotypes derived from “Taishanganying” was approximately 6∶6:6∶0:1∶6 (χ^2^ = 0.132<χ^2^_0.05, 5_ = 11.07; Table 2), which was not significantly different from the theoretical ratio of 1∶1:1∶1:1∶1 (χ^2^ = 9.5<χ^2^_0.05, 5_ = 11.07; Table 2, Table S3 in File S1), although individuals with *S_2_S_4_* were not found in the progeny (Figure S5 in File S1).

Further, individuals with *S_1_S_4_*, *S_1_S_6_*, *S_1_S_2_S_4_*, *S_1_S_2_S_6_*, *S_1_S_4_S_6_*, *S_2_S_4_S_6_* and *S_1_S_2_S_4_S_6_* alleles were found in self-pollinated progeny of “Taishanganying”, but individuals with *S_1_S_2_*, *S_2_S_4_*, *S_2_S_6_* and *S_4_S_6_* were not found in the progeny (Figure S6 in File S1), and their ratio did not fit with the theoretical ratio of 1∶1:1∶1:1∶6:6∶6:6∶6 (χ^2^ = 349.253>χ^2^_0.05, 11_ = 18.31; Table 1, Table S4 in File S1). The ratio of *S_1_S_2_*, *S_1_S_4_*, *S_1_S_6_*, *S_2_S_4_*, *S_2_S_6_* and *S_4_S_6_* alleles was approximately 2∶3:2∶2:1∶2 (χ^2^ = 3.162<χ^2^_0.05, 5_ = 11.07; Table 2), which also did not fit the theoretical ratio of 1∶1:1∶1:1∶1 (χ^2^ = 149.426>χ^2^_0.05, 5_ = 11.07; Table 2). The large number of individuals of *S_1_S_4_* indicated that *S_1_*- and *S_4_*-haplotypes expressed in diploid pollen grains are able to grow better into ovaries than the pollen grains of *S_1_S_6_*, *S_1_S_2_*, *S_2_S_4_*, *S_2_S_6_* and *S_4_S_6_* because of the lowest number of individuals of these *S*-genotypes.

The actual ratio of the single *S*-alleles derived from “Taishanganying”, *S_1_*, *S_2_*, *S_4_* and *S_6_*, was approximately 2∶1:2∶2 (χ^2^ = 2.094<χ^2^_0.05, 3_ = 7.815; Table 2), which was not significantly different from the theoretical ratio of 1∶1:1∶1 (χ^2^ = 5.167<χ^2^_0.05, 3_ = 5.99; Table 2) in cross-pollinated progeny of “Summit”×“Dabai”. But the ratio of *S_1_*, *S_2_*, *S_4_* and *S_6_* alleles was approximately 3∶2:3∶2 (χ^2^ = 2.021<χ^2^_0.05, 3_ = 7.815; Table 2), which did not fit the theoretical ratio of 1∶1:1∶1 (χ^2^ = 301.751>χ^2^_0.05, 3_ = 7.815; Table 2) in self-pollinated progeny of “Taishanganying”. This suggests that the hetero-diploid pollen grains containing the *S_1_*- and/or *S_4_*-haplotype may be able to inactivate stylar S-RNase inside the pollen tube than the other pollen grains.

### Genetic Analysis of *SFB* Alleles in Progenies

The distribution of *Pps-SFB_1_* allele was the same as *Pps-S_1_-RNase* allele in the self-pollinated progeny of “Dabai” (Figure S7 in File S1), *Pps-SFB_5_* was the same as *Pps-S_5_-RNase* allele in the progenies of “Dabai” (Figure S7, S8 in File S1) and *Pps-SFB_4_* allele was the same as *Pps-S_4_-RNase* allele in the progenies of “Taishanganying” (Figure S9, S10 in File S1). While *Pa-SFB_1_* allele was the same as *Pa-S_1_-RNase* allele in the progeny of “Summit”×“Dabai” (Figure S8 in File S1) and *Pa-SFB_2_* allele was the same as *Pa-S_2_-RNase* allele in the progenies of “Summit”×“Dabai” (Figure S8 in File S1) and “Summit”×“Taishanganying” (Figure S9 in File S1). Thus, *SFB* alleles of *P. avium* and *P. pseudocerasus* co-segregated along with their cognate *S-RNases*.

## Discussion

### Analysis of *S-RNase* and *SFB* Alleles

Partial coding sequences of the nine *P. pseudocerasus S-RNase* alleles had previously been cloned from nine Chinese cherry cultivars; these alleles were specifically expressed in the style [44]. The full-length sequence of six of these *S-RNase* alleles have been successfully amplified in this study and found to have high sequence polymorphism. The deduced amino acid sequences showed high sequence identities (70.5 to 79.4%) between these alleles, indicating high polymorphism in the six *S*-allelic sequences. Meanwhile, the presence of five conserved regions (C1, C2, C3, RC4 and C5) and one hypervariable region (RHV; Figure 1) in these alleles indicated that these six *S*-alleles belong to the *Prunus S-RNase* alleles.

Moreover, only four *P. pseudocerasus SFB* alleles with an F-box motif, two variable regions and two hypervariable regions were identified in this study (Figure 2). These *SFBs* were tightly linked to their *S-RNases* (Figure 4), specifically expressed in pollen grains (Figure 3) with high polymorphism (76.9 to 80.0%), and co-segregated along with their cognate *S-RNases* (Figure S7-S10 in File S1), indicating that the four *SFB* alleles belonged to the *Prunus SFB* alleles.

### The *S*-genotype of Pollen Grains in Two Chinese Cherry Cultivars

The ploidy of pollen grains is not identical in different tetraploid species. Diploid pollen grains are produced from tetraploid cultivars in *Lycopersicon peruvianum* [39], *Pyrus sinkiangensis* [29], *Prunus Cerasus* [27], [54]. Monoploid, triploid and/or tetraploid pollen grains are produced from tetraploid cultivars in *Vitis vinifera* [57]. In *Prunus* species, sour cherry and Chinese cherry are the only two tetraploid materials for the study of self-compatibility. The *S*-alleles of tetraploid sour cherry have limited genomic arrangements, for example, self-compatible cultivar ‘Căcănski Rubin’ with phenotype *S_1_S_4_S_13_S_B_* could have the genotype and arrangement *S_1_S_13_.S_4_S_B_* or *S_4_S_13_.S_1_S_B_* that can only produce the pollen grains of *S_1_S_13_*, *S_4_S_B_*, *S_4_S_13_* and *S_1_S_B_* [54]. However, genetic analysis of self-pollinated progeny of Chinese cherry “Nanjingchuisi” suggested that the four *S*-haplotypes complied with tetrasomic inheritance [28]. In the present study, the two interspecific cross-pollinations were performed to characterize the *S*-genotypes of pollen grains derived from two tetraploid Chinese cherry cultivars. The fruit setting of the two interspecific cross-pollinations is low (Table S2 in File S1), maybe due to the significant differences of secretion and surroundings of the stigma between the two species led to the formation of unusual sprouting pollen, pollen tube that could not grow into ovary due to limited velocity and/or length, or abnormal fertilization or embryo development [58]. However, the few seedlings available are enough for study of the *S*-genotype of pollen grains in these two Chinese cherry cultivars. Genetic data of the progeny plants from two interspecies cross-pollinations showed that all individuals contain two different *P. pseudocerasus S*-haplotypes (Table 1), indicating that the pollen grains were hetero-diploid, and that the two *S*-haplotypes were made up of every combination of two of the four *P. pseudocerasus S*-haplotypes in the two tetraploid cultivars, which is different from tetraploid sour cherry [54].

### The Self-compatibility of Tetraploid Chinese Cherry

The breakdown of self-incompatibility can be caused by polyploidy [28], [39] and duplication of the *S*-allele [13], [38]. It is notable that the self-incompatibility of sour cherry was broken down by the accumulation of non-functional *S*-haplotypes [27] but the self-compatibility of Chinese cherry cultivar “Najingchuisi” was caused by competitive interaction [28]. Competitive interaction breaks down self-incompatibility as a consequence of the presence of two *S*-loci of different haplotypes in the pollen grains. In this study, *P. pseudocerasus S_2_*-haplotype is abnormal, with possibly a large sequence insertion or deletion in its *SFB* coding region, causing it to lose its pollen but not style *S*-allele function, like *P. avium SFB_3_^’^* [21]. Conversely, the *P. pseudocerasus SFB_8_* allele that could not be amplified by RT-PCR may result from differential amplification. No mutants leading to the dysfunction of *S_1_*-, *S_4_*-, *S_5_*- or *S_6_*-haplotypes were found based on their full-length cDNA sequences, and the large number of individuals with *S_1_S_5_* and *S_5_S_8_* alleles in the self-pollinated progeny of “Dabai” and individuals with *S_1_S_4_* in the self-pollinated progeny of “Taishanganying” (Table 2) indicate that the hetero-diploid pollen grains were compatible with self styles. Thus, the competitive interaction is responsible for the self-compatibility of Chinese cherry.

### Inheritance of Hetero-diploid Pollen *S*-haplotype

So far, most of the research concerning competitive interaction has been focused on auto-tetraploid and transgenic cultivars where only one hetero-diploid pollen grain exists [38], [39], [59], [60], and the research on polyploid and/or transgenic cultivars where different hetero-diploid pollen grains exist has been minimal [28]. In this study, it is interesting that the hetero-diploid pollen grains produced by the two tetraploid cultivars differ from each other in *S*-haplotype ratios.

The segregation results showed that the ratios of two *S*-alleles expressed in hetero-diploid pollen grains did not fit the theoretical ratios in most progeny plants. In the self-pollinated progeny of “Dabai”, the number of individuals of two *S*-haplotypes (Table 1) indicates that the pollen grains with *S_1_S_5_* and *S_5_S_8_* alleles may have the capability to inactivate stylar S-RNase inside the pollen tube and grow better into ovaries than the pollen grains with *S_1_S_8_*, *S_1_S_2_*, *S_2_S_5_* and *S_2_S_8_* alleles. In the self-pollinated progeny of “Taishanganying”, the number of individuals of two *S*-haplotypes (Table 1) shows that the pollen grains with *S_1_S_4_* allele also may be able to grow better into ovaries in the same way than the pollen grains with *S_1_S_2_*, *S_1_S_6_*, *S_4_S_6_*, *S_2_S_4_* and *S_2_S_6_* alleles. Moreover, the number of individuals containing *S_1_*- and *S_5_*- or *S_8_*-haplotypes were larger than other individuals in the self-pollinated progeny of “Dabai”, and the number of individuals containing *S_1_*- and *S_4_*-haplotypes was greatest in the self-pollinated progeny of “Taishanganying” (Table 2), which shows that the pollen grains with *S_1_S_5_*, *S_5_S_8_* and *S_1_S_4_* alleles have a similar function. Thus, the number of individuals of specific *S*-haplotypes showed that pollen grains with certain combinations could inactivate stylar S-RNase inside the pollen tube and grow better into ovaries than others in each self-pollinated progenies (Table 1).

It is interesting that a few varieties of pollen grains of the same Chinese cherry cultivar are compatible with self-styles; this may be the result of a lethal *P. pseudocerasus S_2_*-haplotype mutant that could induce pollen abortion [61]. Alternateively, diploid pollen grains carrying the non-functional *P. pseudocerasus S_2_*-haplotype are equal to monoploid pollen grains carrying the other functional *S*-haplotype and should therefore have been rejected by self styles [28]. So, the individual containing this *S*-haplotype could hardly be found in the two interspecific cross-pollinated progeny and two self-pollinated progeny plants with two different *S*-alleles. The mechanism that the pollen grains with *S_1_S_5_*, *S_5_S_8_* and *S_1_S_4_* alleles have to grow more efficiently into ovaries than the other pollen grains is unexplainable, but might be a result of the self-incompatibility reaction of pollen grains containing *P. pseudocerasus S_2_*-haplotype, which could moderately restrict the growth of some pollen tubes in compatible mating by reducing support derived from the pistil [62], a long-range activity that could direct some pollen tubes into ovule [63] or a few chemical factors that could regulate some pollen tubes growth, including Ca2+ [64], lipids [65], gibberellins [66] and spermidine [67].

In conclusion, our results demonstrate that the pollen grains of Chinese cherry were hetero-diploid and the two *S*-haplotypes were made up of every combination of two of the four *S*-haplotypes. Some of these hetero-diploid pollen grains result in self-compatibility of Chinese cherry and thus may have the function to inactivate stylar S-RNase inside the pollen tube, by which they can grow better into ovaries than others.

## Supporting Information

File S1 **Supporting information tables and figures.** Table S1 Sequences of primers used in this study. Table S2 Rates of fruit setting in self- or cross-pollination of four cultivars. Table S3 Gamete constitutions in the interspecific cross-pollinated progeny of diploid with hetero-tetraploid plants. Table S4 Gamete constitutions in the self-pollinated progeny of hetero-tetraploid plants. Figure S1 Digestion patterns of PCR amplification products of *SFBs* with six different restriction endonucleases for “Dabai”. M. Ladder marker; 1–23. Digestion patterns of twenty-three independent positive clones. a. Digestion patterns of PCR amplification products with restriction endonuclease DpnII; b. Digestion patterns of PCR amplification products with restriction endonuclease Csp6I; c. Digestion patterns of PCR amplification products with restriction endonuclease HinfI; d. Digestion patterns of PCR amplification products with restriction endonuclease HpyCH4IV; e. Digestion patterns of PCR amplification products with restriction endonuclease BsaJI; f. Digestion patterns of PCR amplification products with restriction endonuclease BslI. Figure S2 Digestion patterns of PCR amplification products of *SFBs* with six different restriction endonucleases for “Taishanganying”. M. Ladder marker; 1–23. Digestion patterns of twenty-three independent positive clones. a. Digestion patterns of PCR amplification products with restriction endonuclease DpnII; b. Digestion patterns of PCR amplification products with restriction endonuclease Csp6I; c. Digestion patterns of PCR amplification products with restriction endonuclease HinfI; d. Digestion patterns of PCR amplification products with restriction endonuclease HpyCH4IV; e. Digestion patterns of PCR amplification products with restriction endonuclease BsaJI; f. Digestion patterns of PCR amplification products with restriction endonuclease BslI. Figure S3 PCR products of *S-RNases* amplified with primers Pru-C2 and Pa-C3R from genomic DNA of two parents (“Summit” and “Dabai”) and some of their cross-pollinated progeny. M. Ladder marker; S. “Summit”; T. “Dabai”; 1–44. Forty-four different individuals. a. Agarose gel electrophoresis patterns of *S-RNase* alleles; b. Polyacrylamide gel electrophoretic patterns of *S-RNase* alleles. Figure S4 PCR products of *S-RNases* amplified with primers Pru-C2 and Pa-C3R from genomic DNA of the parent (“Dabai”) and some of their self-pollinated progeny. M. Ladder marker; T. “Dabai”; 1–45. Forty-five different individuals. Figure S5 PCR products of *S-RNases* amplified with primers Pru-C2 and Pa-C3R from genomic DNA of two parents (“Summit” and “Taishanganying”) and some of their cross-pollinated progeny. M. Ladder marker; S. “Summit”; G. “Taishanganying”; 1–44. Forty-four different individuals. a. Agarose gel electrophoresis patterns of *S-RNase* alleles; b. Polyacrylamide gel electrophoretic patterns of *S-RNase* alleles. Figure S6 PCR products of *S-RNases* amplified with primers Pru-C2 and Pa-C3R from genomic DNA of parent (“Taishanganying”) and some of their self-pollinated progeny. M. Ladder marker; G. “Taishanganying”; 1–45. Forty-five different individuals. Figure S7 PCR products of *SFBs* amplified with primers PsSFB-F1 and PsSFB-R1 from genomic DNA of parent (“Dabai”) and some of their self-pollinated progenies. M. Ladder marker; T. “Dabai”; 1–45. Forty-five different individuals. Figure S8 PCR products of *SFBs* amplified with primers PsSFB-F1 and PsSFB-R1 from genomic DNA of two parents (“Summit” and “Dabai”) and some of their cross-pollinated progenies. M. Ladder marker; S. “Summit”; T. “Dabai”; 1–44. Forty-four different individuals. Figure S9 PCR products of *SFBs* amplified with primers PsSFB-F1 and PsSFB-R1 from genomic DNA of two parents (“Summit” and “Taishanganying”) and some of their cross-pollinated progenies. M. Ladder marker; S. “Summit”; G. “Taishanganying”; 1–21. Forty-four different individuals. Figure S10 PCR products of *SFBs* amplified with primers PsSFB-F1 and PsSFB-R1 from genomic DNA of parent (“Taishanganying”) and some of their self-pollinated progenies. M. Ladder marker; G. “Taishanganying”; 1–45. Forty-five different individuals.
(DOC)
