# Supplementary material for: Inheritance of Hetero-Diploid Pollen S-Haplotype in Self-Compatible Tetraploid Chinese Cherry (Prunus pseudocerasus Lindl)
Source: PLoS One. 2013 Apr 15;8(4):e61219. doi: 10.1371/journal.pone.0061219 (PMC3626605; doi:10.1371/journal.pone.0061219)

**Supporting Tables:**

**Table S1** Rates of fruit setting in self- or cross-pollination of four cultivars

| Self- or Cross- pollination | | Number of flowers | Number of set fruits | Fruit setting (%) |
| --- | --- | --- | --- | --- |
| Self-pollination | ‘DB’ | 5212 | 1013 | 19.43 |
|  | ‘TG’ | 3692 | 745 | 20.18 |
|  | ‘SMT’ | 1073 | 3 | 0.28 |
| Cross-pollination | ‘SMT’× ‘DB’ | 2815 | 58 | 2.06 |
|  | ‘SMT’×‘TG’ | 2106 | 30 | 1.42 |

**Table S2** Primers for PCR analysis of *S-RNase* and *SFB* genes in Chinese cherry.

| Primer name | Sequence (5’ to 3’) | Length (bp) | Note |
| --- | --- | --- | --- |
| Pru-C2 | CTATGGCCAAGTAATTATTCAAACC | 25 | Tao et al. (1999) |
| Pa-C3R | TTGTATCATTGCCACTTTCCACG | 23 | Gu et al. (2010) |
| Pa-C5R | CAAAATACCAC TTCATGTAGCAACTG | 26 | Gu et al. (2010) |
| GC-C3F-1 | CGTGGAAAGTGGCAATGATAC | 21 | Gu et al. (2011) |
| GC-C3F-2 | TGGGAAAGCGAATGGAACAAACAT | 24 | Gu et al. (2011) |
| Pa-C4F | TTACANANATCCTTAAAAACGCTTCAAT | 28 | Used in this study |
| PsSFBF1 | GAAAWCKTAATCGACATCCTMGTAAG | 26 | Zhang et al. (2007) |
| PsSFBR1 | CAMRAATTCGATTTCGYCATATTTC | 25 | Zhang et al. (2007) |
| Mid-SFB-1 | AACAGACTCTTGGAAGATGATT | 22 | Gu et al. (2011) |
| Mid-SFB-2 | TCCTCCTTGGTTAAAATGCACTTGG | 25 | Gu et al. (2011) |
| Pps-S8R | CTGAGTCTTCGCTTGGCCCTTAG | 23 | Gu et al. (2010) |
| ActF | CAATGTGCCTGCCATGTATG | 20 | Zhang et al. (2007) |
| ActR | CCAGCAGCTTCCATTCCAAT | 20 | Zhang et al. (2007) |

Note: Y = C/T; S = C/G; W = A/T; N = G/A/T/C.

**Table S3** Gamete constitutions in the interspecific cross-pollinated progeny of diploid with hetero-tetraploid plants

| Pollinator | | Hetero-tetraploidy (*S_a_S_b_ S_c_S_d_*) | | | | | |
| --- | --- | --- | --- | --- | --- | --- | --- |
| Receptor | gamete | *S_a_S_b_* | *S_a_S_c_* | *S_a_S_d_* | *S_b_S_c_* | *S_b_S_d_* | *S_c_S_d_* |
| Diploidy  (*S_1_S_2_*) | *S_1_* | *S_a_S_b_*/*S_1_* | *S_a_S_c_*/*S_1_* | *S_a_S_d_*/*S_1_* | *S_b_S_c_*/*S_1_* | *S_b_S_d_*/*S_1_* | *S_c_S_d_*/*S_1_* |
|  | *S_2_* | *S_a_S_b_*/*S_2_* | *S_a_S_c_*/*S_2_* | *S_a_S_d_*/*S_2_* | *S_b_S_c_*/*S_2_* | *S_b_S_d_*/*S_2_* | *S_c_S_d_*/*S_2_* |

Note: If no classes of pollen are rejected, the ratio of zygote with any of *S*-genotype are 1/12, the ratio of zygote containing any of *S*-haplotype of receptor (*S_1_* and *S_2_*) are 6/12, and the ratio of zygote contianing any of *S*-haplotype combinations of pollinator (*S_a_S_b_*, *S_a_S_c_*, *S_a_S_d_*, *S_b_S_c_*, *S_b_S_d_* and *S_c_S_d_*) are 2/12.

**Table S4** Gamete constitutions in the self-pollinated progeny of hetero-tetraploid plants

| gamete | *S_a_S_b_* | *S_a_S_c_* | *S_a_S_d_* | *S_b_S_c_* | *S_b_S_d_* | *S_c_S_d_* |
| --- | --- | --- | --- | --- | --- | --- |
| *S_a_S_b_* | *S_a_S_b_* | *S_a_S_b_S_c_* | *S_a_S_b_S_d_* | *S_a_S_b_S_c_* | *S_a_S_b_S_d_* | *S_a_S_b_S_c_S_d_* |
| *S_a_S_c_* | *S_a_S_b_S_c_* | *S_a_S_c_* | *S_a_S_c_S_d_* | *S_a_S_b_S_c_* | *S_a_S_b_S_c_S_d_* | *S_a_S_c_S_d_* |
| *S_a_S_d_* | *S_a_S_b_S_d_* | *S_a_S_c_S_d_* | *S_a_S_d_* | *S_a_S_b_S_c_S_d_* | *S_a_S_b_S_d_* | *S_a_S_c_S_d_* |
| *S_b_S_c_* | *S_a_S_b_S_c_* | *S_a_S_b_S_c_* | *S_a_S_b_S_c_S_d_* | *S_b_S_c_* | *S_b_S_c_S_d_* | *S_b_S_c_S_d_* |
| *S_b_S_d_* | *S_a_S_b_S_d_* | *S_a_S_b_S_c_S_d_* | *S_a_S_b_S_d_* | *S_b_S_c_S_d_* | *S_b_S_d_* | *S_b_S_c_S_d_* |
| *S_c_S_d_* | *S_a_S_b_S_c_S_d_* | *S_a_S_c_S_d_* | *S_a_S_c_S_d_* | *S_b_S_c_S_d_* | *S_b_S_c_S_d_* | *S_c_S_d_* |

Note: If no classes of pollen are rejected, the ratio of zygote with *SaSb*, *SaSc*, *SaSd*, *SbSc*, *SbSd* and *ScSd* alleles are 1/36, and the ratio of zygote with *SaSbSc*, *SaSbSd*, *SaScSd*, *SbScSd* and *SaSbScSd* alleles are 6/36.

**Supporting figures**

Figure S1


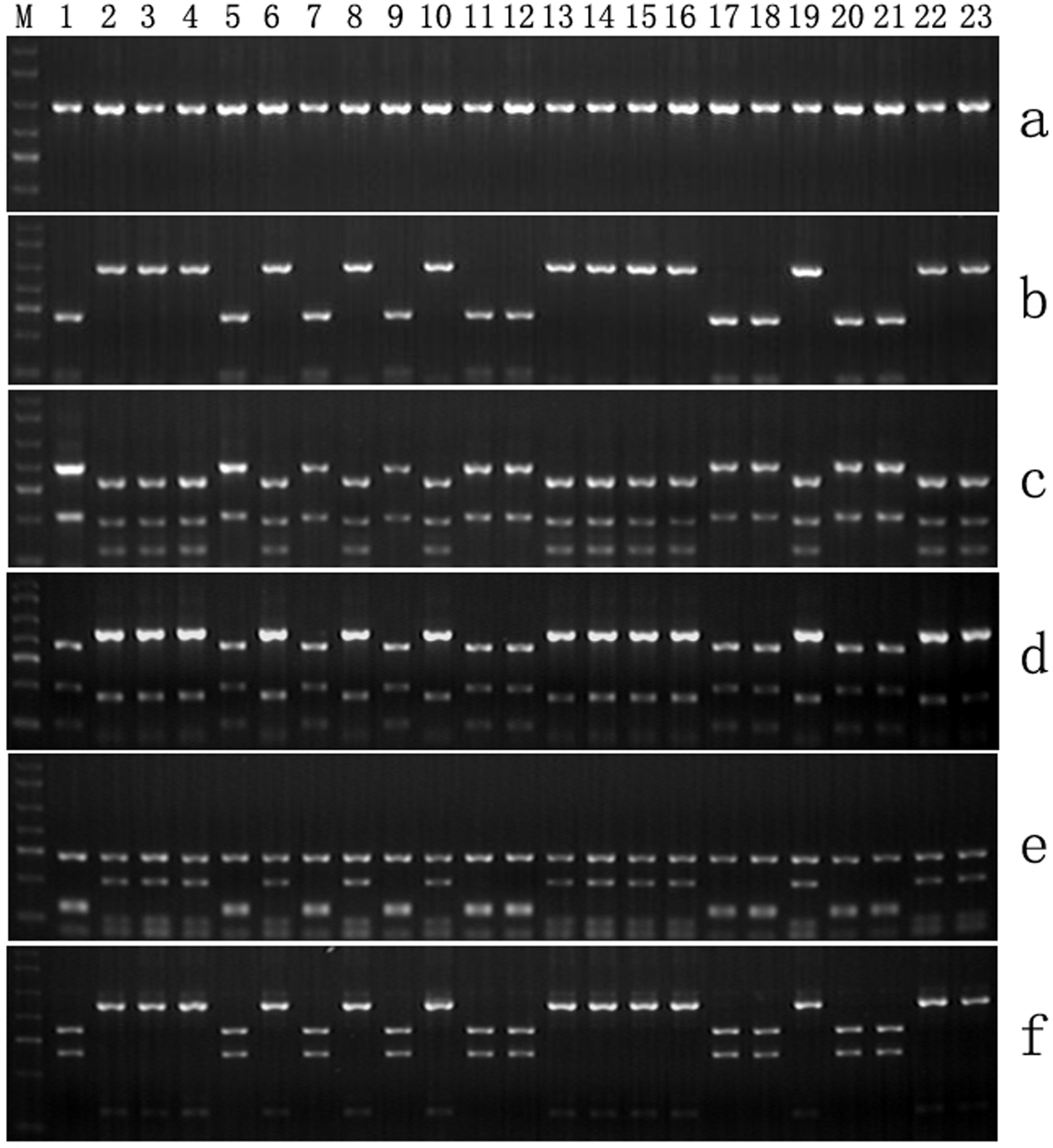


Figure S2


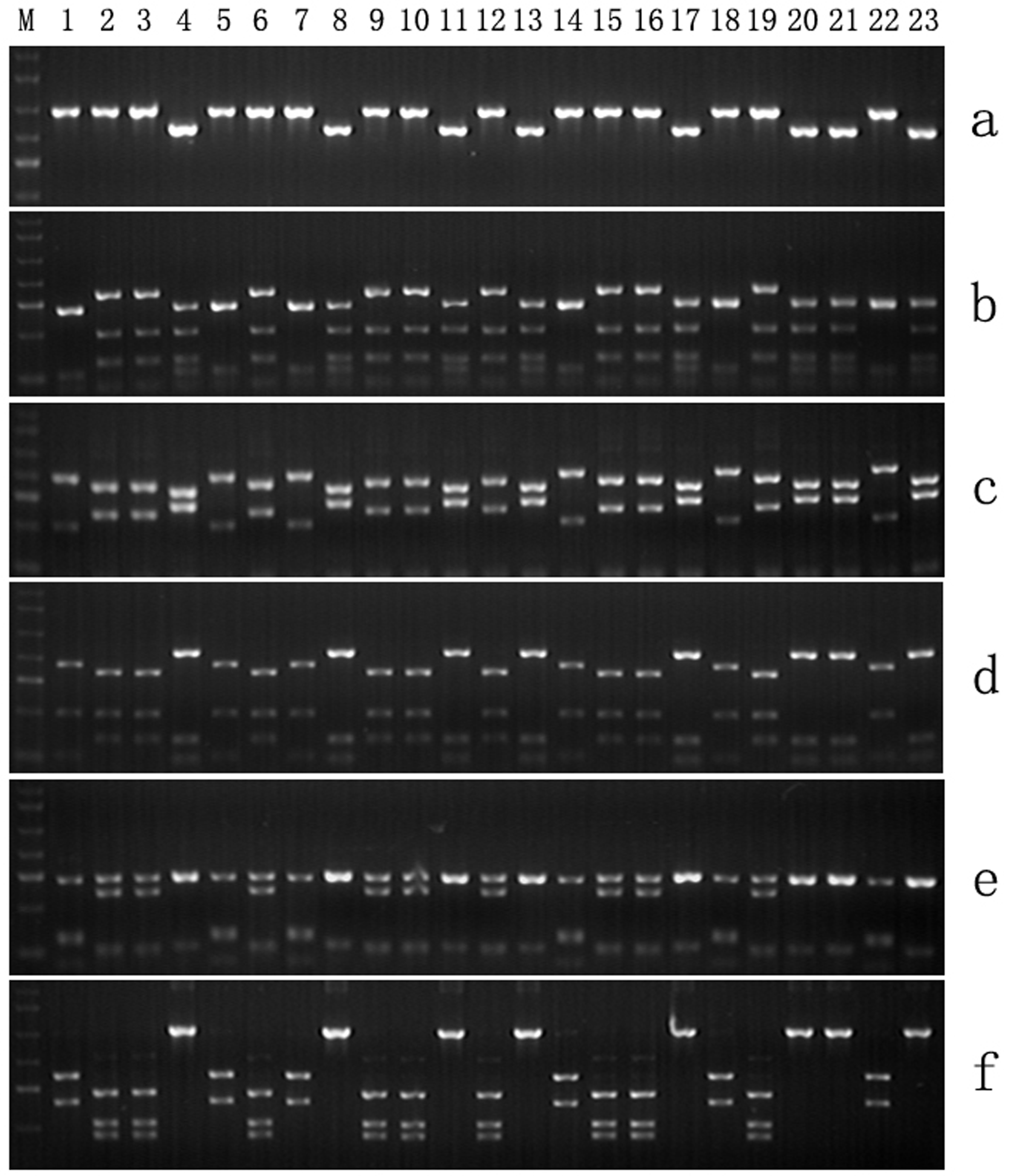


Figure S3


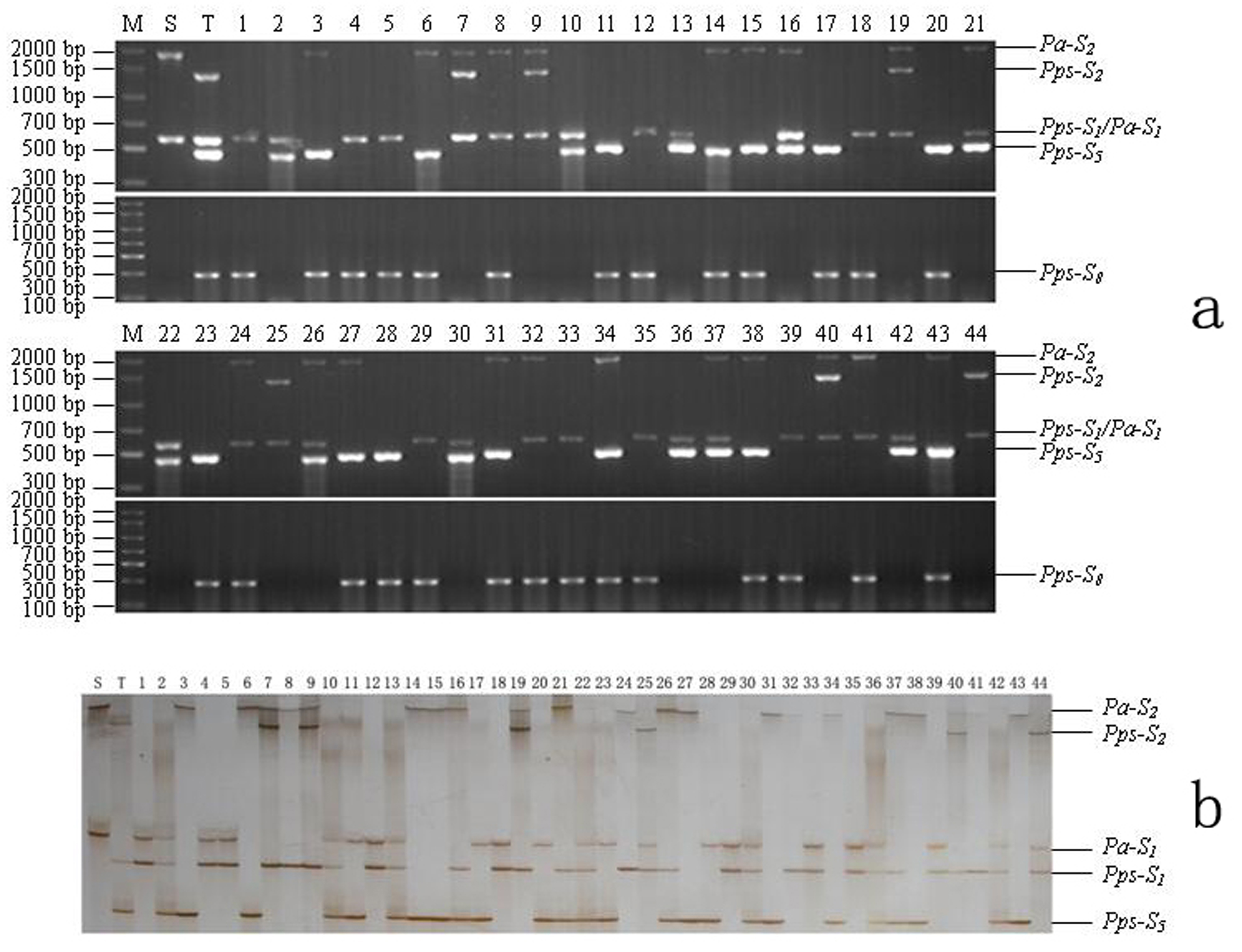


Figure S4


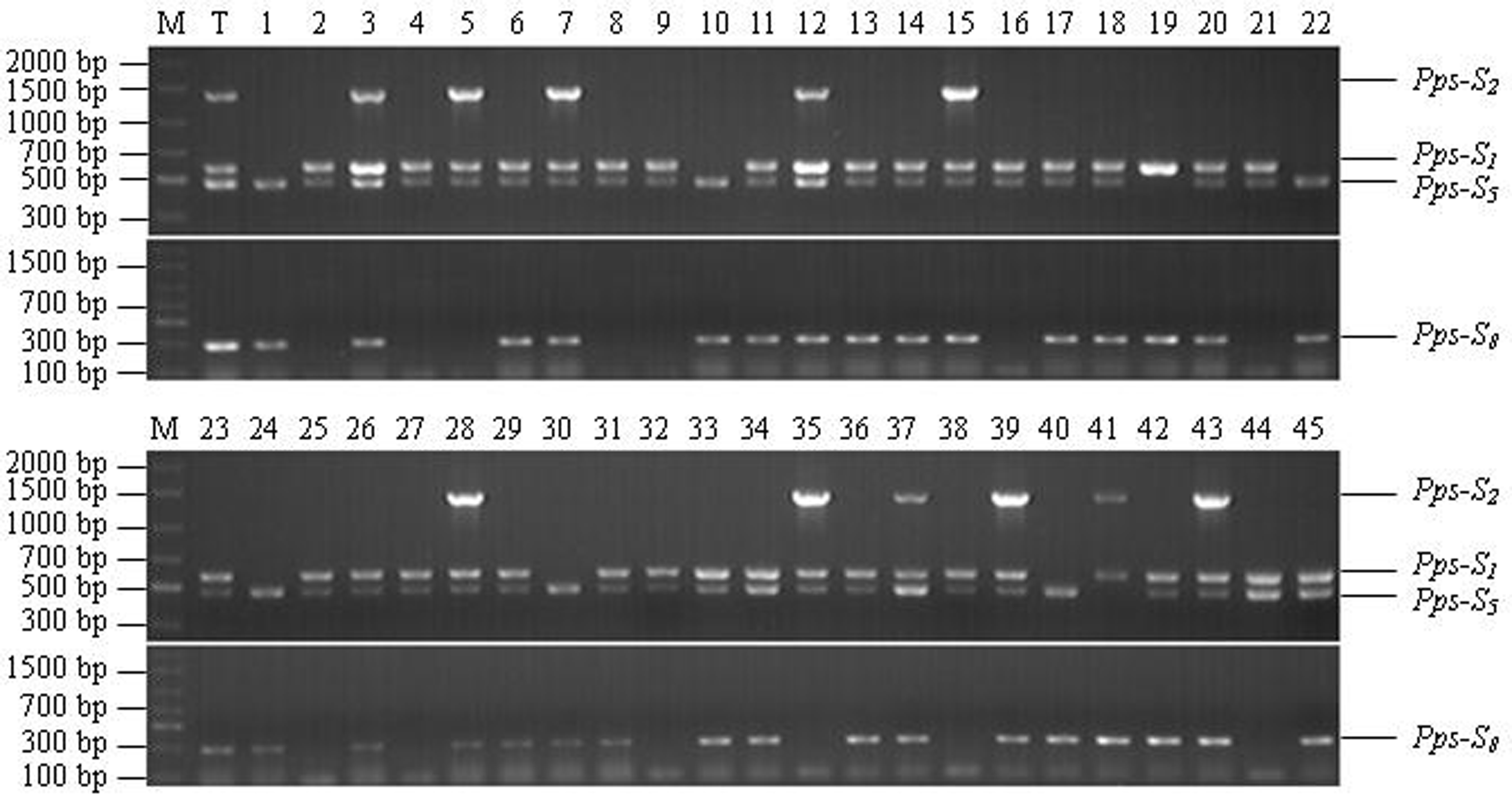


Figure S5


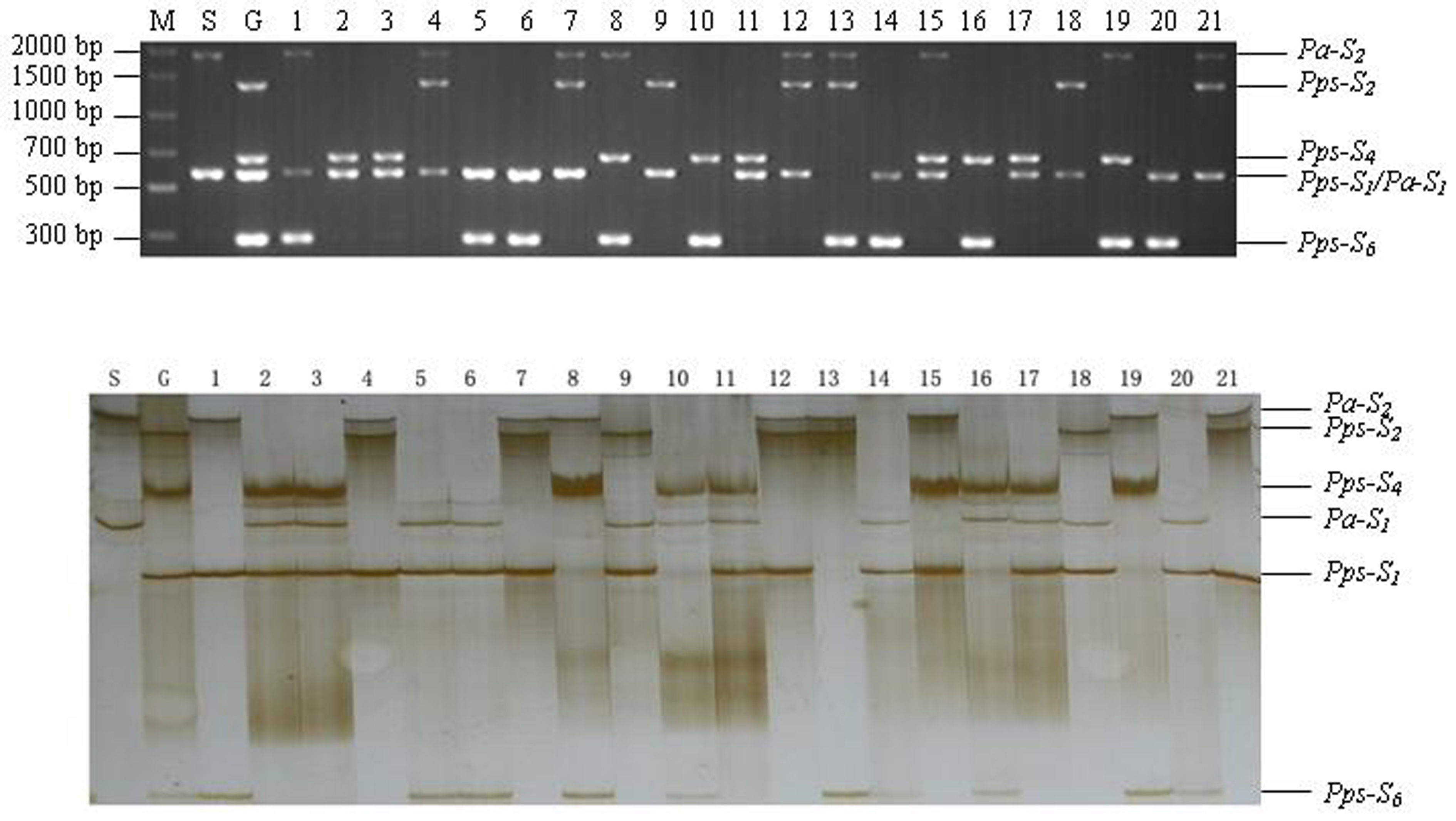


Figure S6


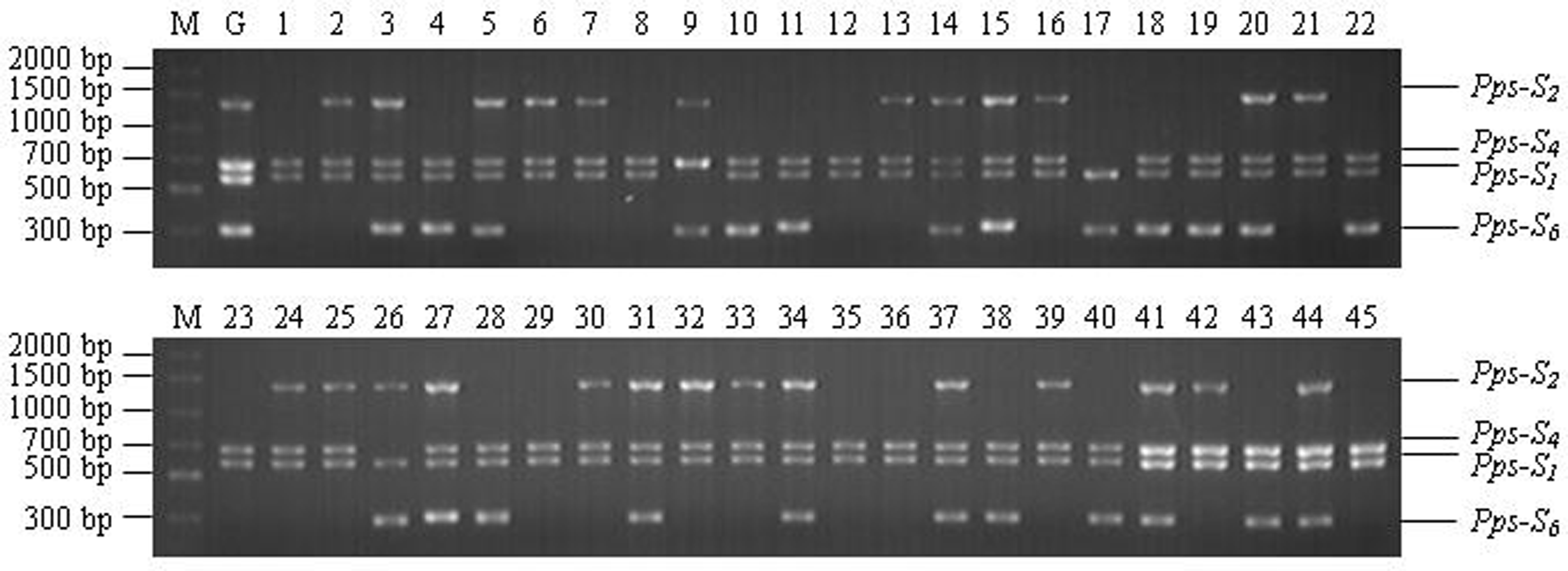


Figure S7


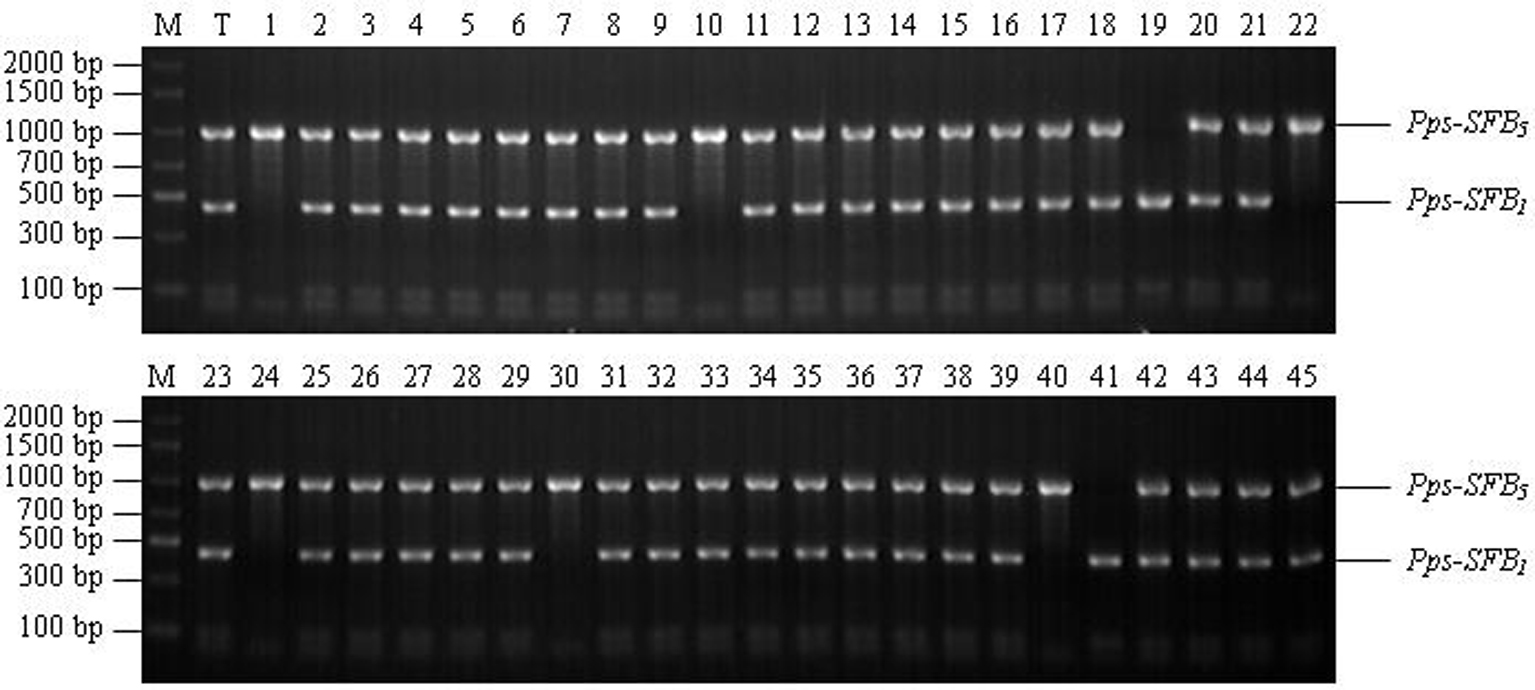


Figure S8


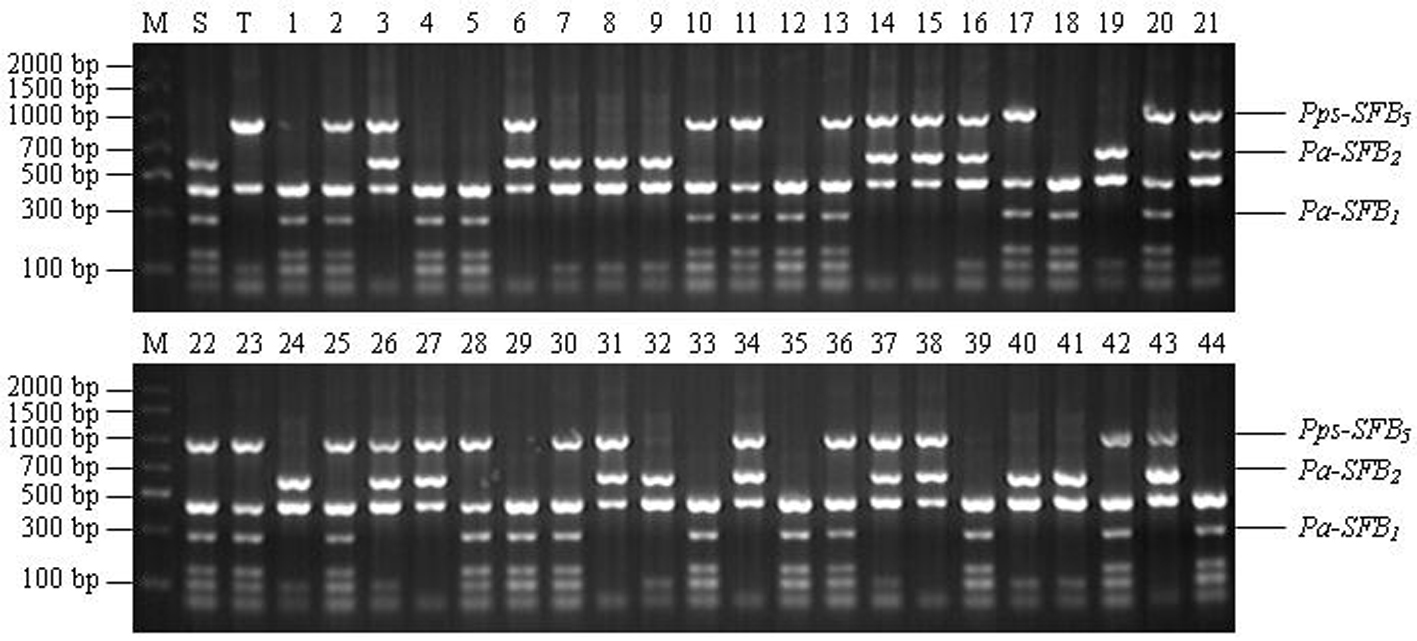


Figure S9


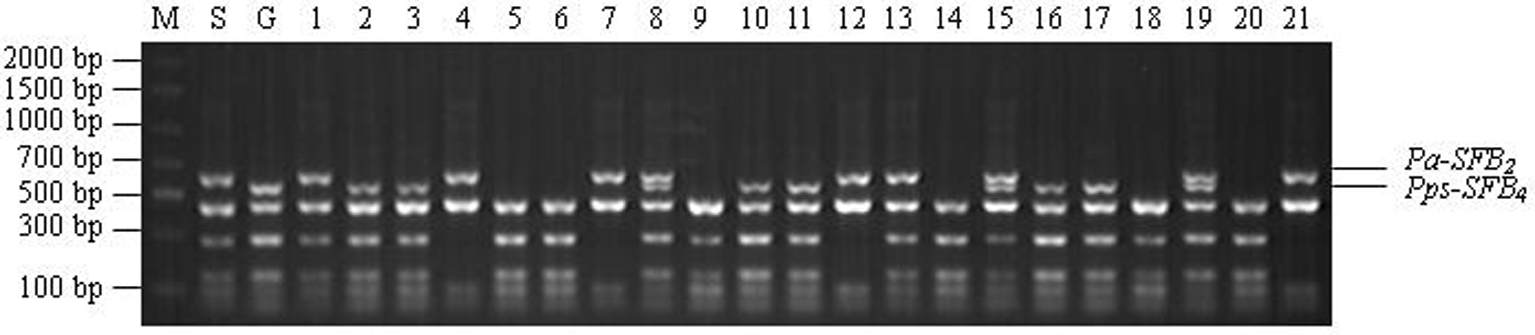


Figure S10


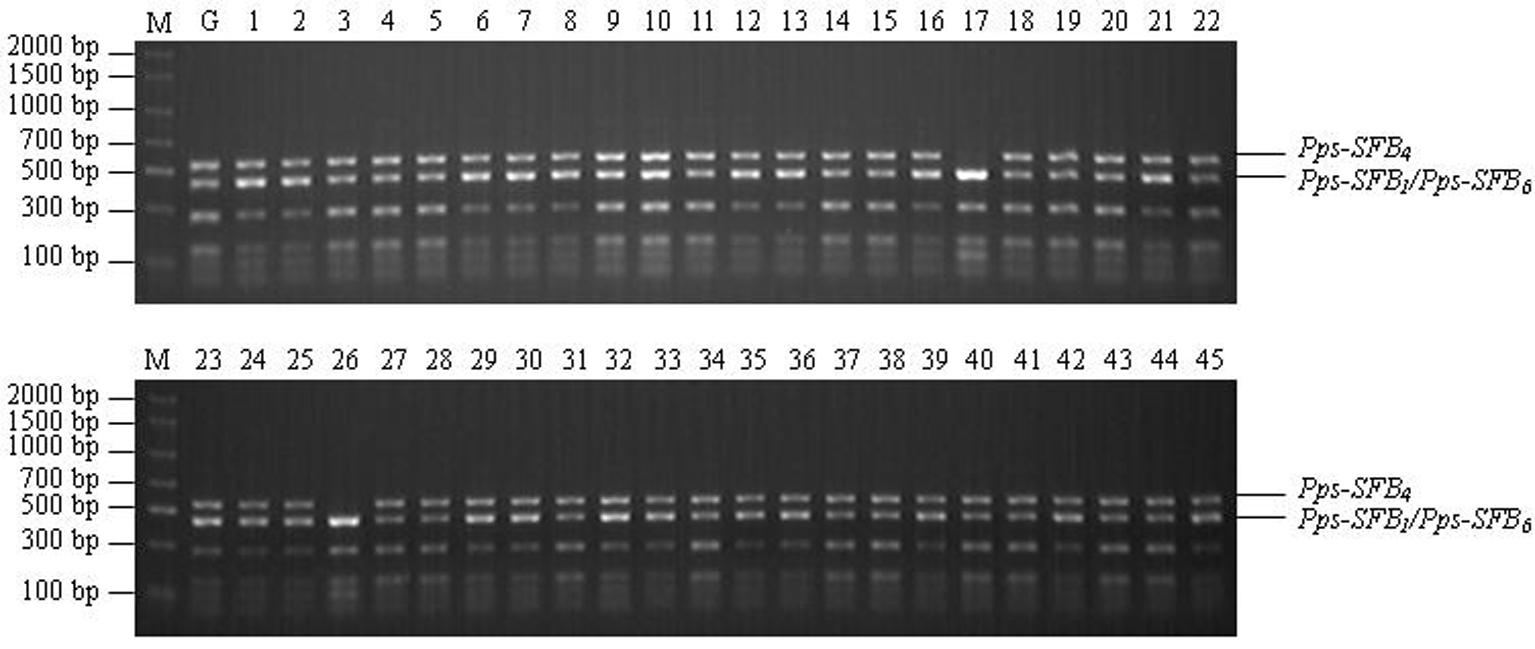

Supplement: File S1 — Supporting information tables and figures. Table S1 Sequences of primers used in this study. Table S2 Rates of fruit setting in self- or cross-pollination of four cultivars. Table S3 Gamete constitutions in the interspecific cross-pollinated progeny of diploid with hetero-tetraploid plants. Table S4 Gamete constitutions in the self-pollinated progeny of hetero-tetraploid plants. Figure S1 Digestion patterns of PCR amplification products of SFBs with six different restriction endonucleases for “Dabai”. M. Ladder marker; 1–23. Digestion patterns of twenty-three independent positive clones. a. Digestion patterns of PCR amplification products with restriction endonuclease DpnII; b. Digestion patterns of PCR amplification products with restriction endonuclease Csp6I; c. Digestion patterns of PCR amplification products with restriction endonuclease HinfI; d. Digestion patterns of PCR amplification products with restriction endonuclease HpyCH4IV; e. Digestion patterns of PCR amplification products with restriction endonuclease BsaJI; f. Digestion patterns of PCR amplification products with restriction endonuclease BslI. Figure S2 Digestion patterns of PCR amplification products of SFBs with six different restriction endonucleases for “Taishanganying”. M. Ladder marker; 1–23. Digestion patterns of twenty-three independent positive clones. a. Digestion patterns of PCR amplification products with restriction endonuclease DpnII; b. Digestion patterns of PCR amplification products with restriction endonuclease Csp6I; c. Digestion patterns of PCR amplification products with restriction endonuclease HinfI; d. Digestion patterns of PCR amplification products with restriction endonuclease HpyCH4IV; e. Digestion patterns of PCR amplification products with restriction endonuclease BsaJI; f. Digestion patterns of PCR amplification products with restriction endonuclease BslI. Figure S3 PCR products of S-RNases amplified with primers Pru-C2 and Pa-C3R from genomic DNA of two parents (“S [file pone.0061219.s001.doc]
